# The Effect of Localized Vibration during Welding on the Microstructure and Mechanical Behavior of Steel Welds

**DOI:** 10.3390/ma12162553

**Published:** 2019-08-10

**Authors:** Ehud Ingram, Oz Golan, Rami Haj-Ali, Noam Eliaz

**Affiliations:** 1 School of Mechanical Engineering, Tel-Aviv University, Ramat Aviv, Tel Aviv 6997801, Israel; 2 School of Mechanical Engineering, Afeka-Tel-Aviv Academic College of Engineering, Tel Aviv 6997801, Israel; 3 Department of Materials Science and Engineering, Tel-Aviv University, Ramat Aviv, Tel Aviv 6997801, Israel

**Keywords:** vibratory weld conditioning, local vibrational effect (LVE), global vibrational effect (GVE), vibratory stress relief (VSR), rigid-body motion, digital image correlation (DIC)

## Abstract

The use of vibratory welding is treated with some caution in the industry due to inconsistent beneficiary results. Here, a partial explanation is suggested by the differentiation between global vibrational effects (GVEs) and local vibrational effects (LVEs), and the latter is investigated experimentally. Two structural plates of steel are welded at three frequency/amplitude combinations using manual gas metal arc welding in an experimental setup that ensures only LVEs. After welding, tensile tests, microhardness tests, and metallurgical characterization are performed locally in the different welding zones and the results are compared to the non-vibrated welds. Novel use of digital image correlation (DIC) is implemented in tensile testing of welded samples, thus enabling the separate determination of local mechanical properties of the base metal, heat-affected zone and fusion zone of the same weld. LVE is found not to promote any distinct difference in weld properties, at least within the vibrational regimes studied. Nevertheless, depending on geometry and structural response, it is explained how vibratory welding may promote residual stress relief due to GVEs of the welded structure.

## 1. Introduction

There are many oscillation-involved welding processes, including weld-pool stirring by oscillations of an electromagnetic field or external dipped electrode, ultrasonic agitation of the weld pool via an external transducer, arc oscillations by interchanging electromagnetic field or by oscillating the weld electrode, etc. [1,2,3,4,5,6]. All of these methods have two things in common: (1) The effect on the weld is local, concentrating on vibrating the weld pool as it solidifies with virtually no effect on the surrounding part; (2) these methods require special equipment and cannot be easily implemented with of-the-shelf welding equipment. Therefore, the appeal of a method that uses conventional welding equipment with the addition of only an external vibrator to achieve improvement of weld quality is clear. Results of numerous works have not been consistent enough to yield a “recipe” or a standardized process for producing favorable welds using external vibration. Theoretically, we can induce any pre-defined combination of vibration parameters (e.g., frequency and amplitude in a certain direction) to a welded joint. However, in reality, when attempting to vibrate structures of complex geometry weighing between tens of kilograms and several tons, it is practically impossible to induce a rigid-body-motion vibration in a desired pre-defined frequency/amplitude/direction due to the vibrator physical size, power limitations, and the effect of the structural response.

The underlying principle of commercial vibrators for welding purposes, as well as for vibratory stress relief (VSR) applications, is that with relatively portable equipment of a reasonable size, it is possible to harmonically excite the structure at resonant or near-resonant frequencies to produce relatively large displacements. This excitation of the structure has a dual effect on the weld, depending on the joined parts. Let us distinguish between local (micro) and global (macro) effects of vibration acting on the vibrated welded joint as part of a structure, assuming all other welding variables are constant.

**Figure 1:**
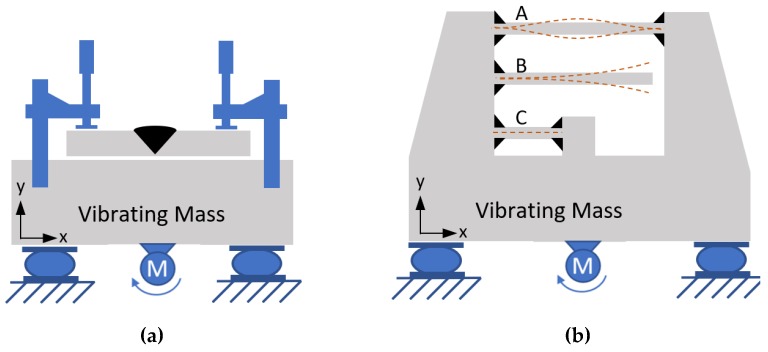
Local vibrational effect (LVE) setup vs. global vibrational effect (GVE) setup. (**a**) A weld coupon is clamped to a vibrator, which is supported by dampers. The weld joint area will experience the same rigid-body motion as the vibrator (LVE). (**b**) A large infinitely-rigid base to which three beams are welded. Assuming the same-section properties, the natural frequencies of the three beams are quite different, “B” having the lowest frequency, “C” having the highest, and “A” in between.

Local vibration effects (LVEs) are the effects that the vibrational parameters (e.g., acceleration, frequency, direction) have on the solidifying molten weld pool as it propagates along the path of the weld line. In other words, it is the rigid-body-motion vibration of the localized weld pool and heat-affected zone (HAZ). An example of LVE setup is shown in Figure 1a. The local effects may influence the diffusion rate, heat convection rate, and temperature gradient at the boundaries of the melt pool caused by the vibrational stirring of the liquid and shear stresses applied on the liquid (or semi-liquid) by the translating/vibrating base-metal walls, which are also affected by the joint preparation geometry. This case is similar in its geometrical boundary conditions to vibrational castings (but not in terms of heat transfer).

Global vibrational effects (GVEs) are the effects that the weld joint experiences while it is connected to a vibrating structure. These global effects due to structural response occur for any real part with finite rigidity. A schematic example of a setup that allows for GVE is shown in Figure 1b. Parts “A”, “B” and “C” will experience these GVEs depending on their stiffness ratio. These parts of the structure will experience both LVEs and GVEs, and the vibrating members will induce interchanging tension-compression stresses in the joint. These stresses may exceed the yield strength, especially when the base material is heated due to the welding operation and the yield strength is low. This means that weld solidification and HAZ cooling take place under interchanging stresses.

From an empirical perspective, in order to conclude which parameters induce a change in weld properties, it is crucial to break this coupling of GVE and LVE and examine the effects separately.

The objective of this work is to examine the effectiveness of low-frequency vibration in rigid-body-motion mode (LVE) in producing mechanical and/or microstructural change in structural steel welds. This is achieved by an experimental setup that ensures only LVE of the weld, without flexural motion of the joint (GVE), and by implementing vibrational welding at several frequencies and amplitudes for comparison.

## 2. Materials and Methods

Two common structural plates of steel were selected: EN 10025 S235JR and ASTM A516 Gr70. These materials were selected due to their widespread use in industry for manufacturing of steel structures.

**Figure 2:**
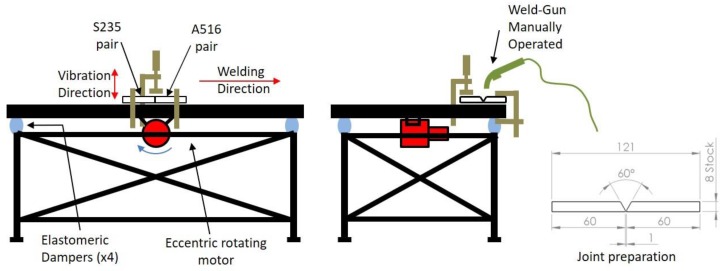
Schematic weld table setup. For each frequency, both pairs of samples (S235 and A516 steels) were welded at the same pass. On the right: The geometry of joint preparation.

Samples of 60 × 120 × 8 mm plates were welded using AWS A5.18 ER70S6 ø1 mm solid wire with a single-V groove joint preparation. Pairs of the same steel were welded by gas metal arc welding (GMAW) with shielding gas of 80%Ar–20%CO_2_ in a single-pass weld to avoid thermal effects typical of multi-pass welds. For each frequency, two plate mates were welded consecutively in the same pass. The welding speed was calculated by timing the weld pass duration and dividing it by the length of the adjoined plates. Welding was performed on a special welding table, designed for vibratory welding and consisting of a thick plate mounted on four elastomeric pads with an eccentric motor fixed to the bottom of the table, as shown in Figure 2. The vibratory system used was a PPAW 5500 Meta-Lax/Bonal system (Royal Oak, MI, USA).

**Figure 3:**
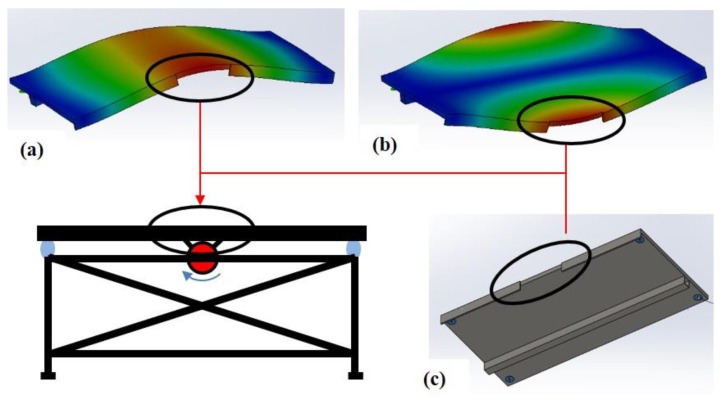
Vibration mode analysis. (**a**) Frequency analysis, 1^st^ mode, qualitative deformation map. (**b**) Frequency analysis, 2^nd^ mode, qualitative deformation map. (**c**) CAD model for FEA of the welding table plate, simulating elastomer pads at the four corners.

The vibration of the plate (or any other geometry) can produce stagnant spatial areas that do not translate, depending on the mode of vibration. In order to ensure definitive local translation regardless of frequency, a preliminary frequency analysis was performed. A 3D computer aided design (CAD) model of the welding table was prepared. Frequency analysis using finite element analysis (FEA) was performed with four elastomeric fixtures at the plate’s corners. Based on these qualitative results, the specific location for test coupon placement was determined, in an area that vibrates throughout the first few modes of vibration, as shown in Figure 3.

The table was vibrated while measuring its vibrations. A metal cube with two fixed accelerometers (100 mV/g Endevco 41A (Sunnyvale, CA, USA) was fixed to the table surface at the location of the welded samples. The accelerometers were connected to a National Instruments data acquisition system (Austin, TX, USA) with 24-bit resolution over ± 50 g input using a sampling rate of 500 samples per second. The system was excited with shock responses (hammer strike) on each axis as well as continuous variable excitation in order to map its resonant frequency and find the frequencies that induce maximal amplitudes at the specific measured location. The frequencies and amplitudes obtained by the structural response as well as the welding parameters are summarized in Table 1.

**Figure 4:**
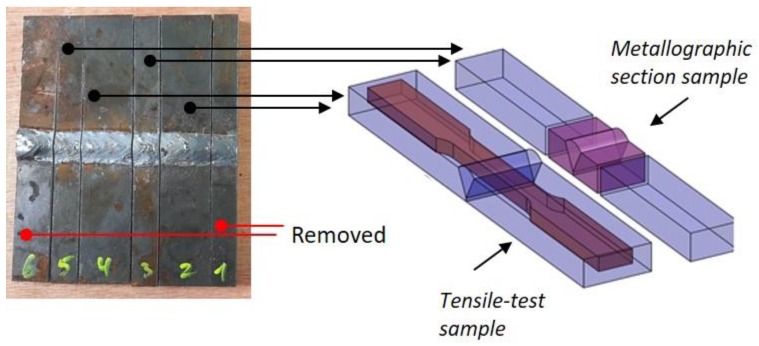
Sample preparation for metallurgical and mechanical characterization. Note the relative position of the FZ.

The samples were trimmed at the edges to remove transient effects of the start and endpoints of the welding seam. Each sample was transversely cut to strips, to produce tensile-test specimens and metallographic cross-sections (Figure 4). Metallographic cross-sections revealing the base metal (BM), HAZ and fusion zone (FZ) were mechanically ground and polished and etched using 3% Nital according to ASTM E407-07. The etched samples were examined under a light microscope (Nikon Eclipse MA200, Tokyo, Japan) and a low-vacuum scanning electron microscope (LV-SEM, Tescan Vega-3LM, Brno-Kohoutovice, Czech Republic) equipped with an Oxford energy-dispersive X-ray spectrometer (EDS) for elemental analysis. Vickers microhardness measurements were conducted on the same metallurgical cross-sections according to ASTM E384-02, using a FutureTech FM-700e machine (Kawasaki-City, Japan) and applying a load of 1 kgf for 15 s.

Specimens for tension tests with digital image correlation (DIC) were machined to ASTM E8-11 sub-size specimens. The samples were coated with contrasting speckles and tested in an INSTRON 5582 machine (Norwood, IL, USA) to which a LaVision system with Elite-2M camera (Göttingen, Germany) and Fujinon FH-25-HA (Minato-ku, Japan) lens was attached for DIC. The tests were conducted at a strain rate of 2 mm/min, a frame rate of 1 fps, and a calibrated resolution of 29.516 μm/pixel. DIC post-processing was done using DaVis 8.2.0 software package.

## 3. Results

### 3.1. Microstructure

**Figure 5:**
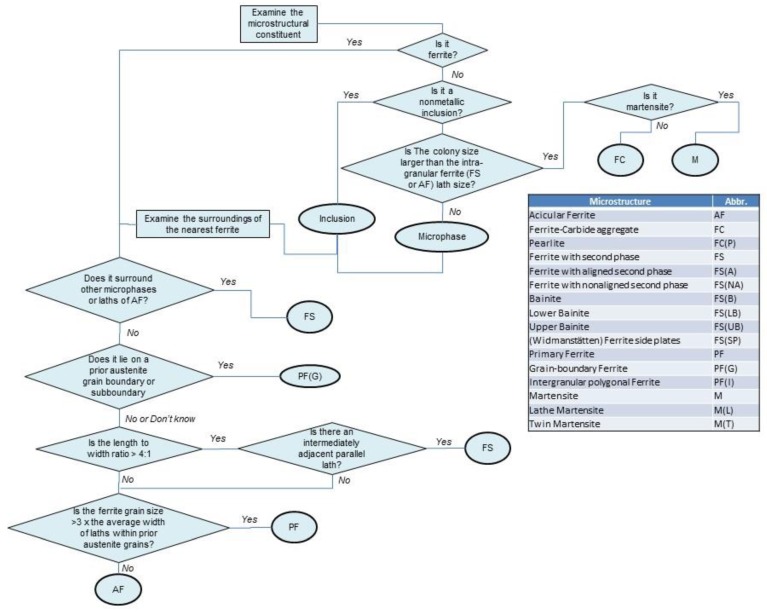
Classification of microstructures [8]. Courtesy of the IIW—International Institute of Welding © IIW.

The classification of microstructures herein follows the International Institute of Welding (IIW) terminology [8], as shown in Figure 5.

**Figure 6:**
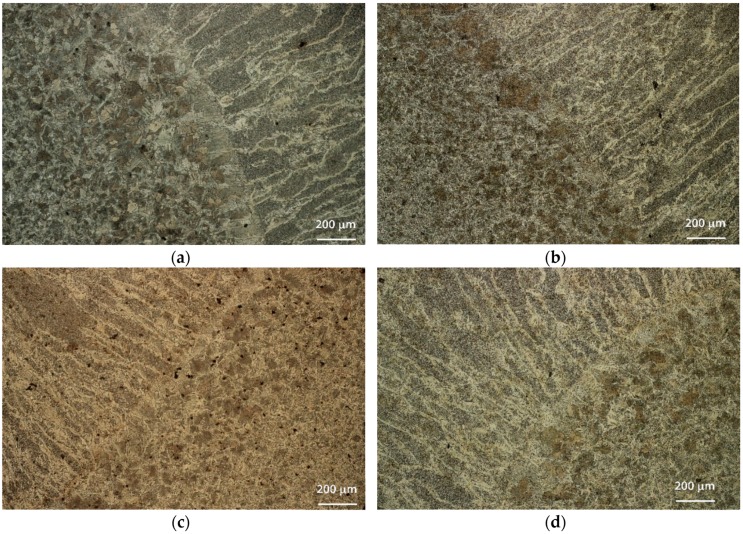
Comparative metallurgical cross-sections through the fusion zone and heat-affected zone of S235 steel, at different vibration frequencies: (**a**) No vibration, (**b**) 12.8 Hz, (**c**) 58.4 Hz, (**d**) 60 Hz.

**Figure 7:**
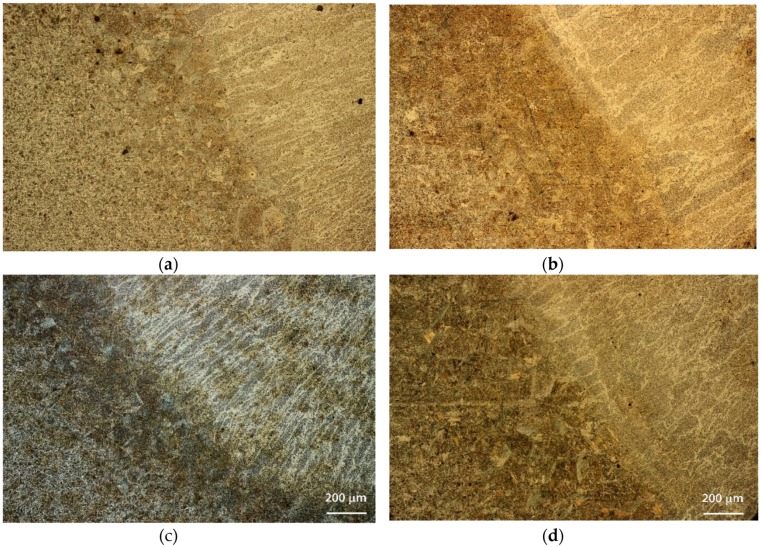
Comparative metallurgical cross-sections through the fusion zone and heat-affected zone of A516 steel, at different vibration frequencies: (**a**) No vibration, (**b**) 12.8 Hz, (**c**) 58.4 Hz, (**d**) 60 Hz.

**Figure 8:**
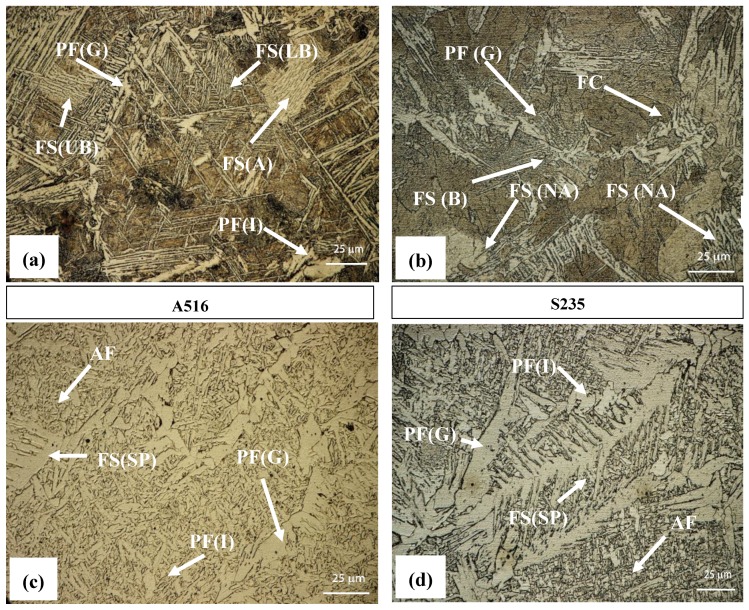
Coarse-grains heat-affected zone (CGHAZ) (**a**,**b**) and fusion zone (**c**,**d**) of A516 Gr. 70 steel (**a**,**c**) and S235 steel (**b**,**d**). Terminology as in Figure 5.

The metallographic characterization revealed no distinct difference between the vibrated samples and the non-vibrated samples. Typical microstructures, focusing on the boundary area between the FZ and HAZ, are shown in Figure 6 and Figure 7 for the S235 steel weld and the A516 steel weld, respectively. All fusion zones (at all frequencies) exhibited directional solidification grains, in the direction perpendicular to the pool boundary where the temperature gradient was maximal. The vibrated samples did not exhibit any change in this directional microstructure. The acicular ferrite content in the FZ was in the range of 60–70%, regardless of the steel type and welding frequency. No noticeable effect of vibration was evident. Acicular ferrite is a desired microstructure, allowing high impact toughness. All samples had similar microstructures in the HAZ, regardless of vibration variables. Typical microstructures of the FZ and coarse-grains HAZ (CGHAZ) are shown in Figure 8.

### 3.2. Microhardness

**Figure 9:**
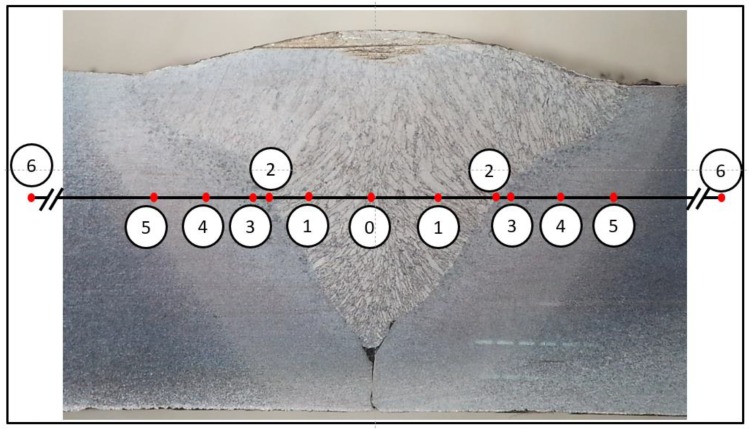
Position of different microhardness indents along the scan line and the corresponding microstructure.

**Figure 10:**
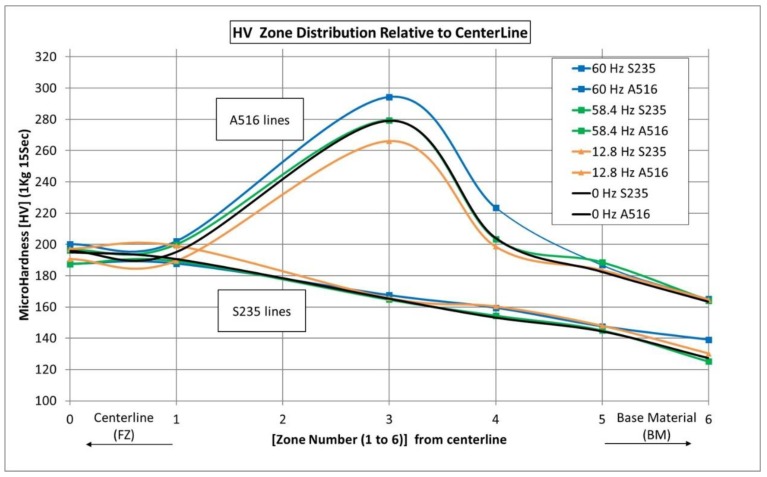
Microhardness values along the scan line shown in Figure 9. Each point on this graph represents the average of four sampled points.

For each steel welded under different vibrational conditions, two specimens were tested (16 in total). Since both the FZ boundaries and the HAZ width vary slightly in different cross-sections, a corrected microstructural-based distribution is suggested in Figure 9. Indentations were made at these positions. The results were plotted with the abscissa representing the different regions (from the centerline outwards), thus allowing for the application of a symmetry condition. Excluding point “2” (an attempt to indent the boundary line between the FZ and HAZ), the corrected data is presented in Figure 10. Each point on this graph represents the average of four sampled points.

The microhardness results are within the expected values for these types of steels and filler wire. No distinct difference was evident between vibrated and non-vibrated samples, and no trend was observed as the vibration frequency was raised. The welded samples of S235 steel had an average peak hardness of 195 VHN at the FZ, and moderately descending hardness values away from the FZ, down to an average of 130 VHN at the BM. In comparison, the A516 Gr. 70 welded steel samples exhibited an average hardness of 195 VHN at the FZ, and steep ascend to an average of 280 VHN in the CGHAZ adjacent to the fusion line. The hardness values, in this case, descended further away from the FZ, down to an average of 165 VHN at the BM, with very small deviations from this value.

### 3.3. Tensile Tests with DIC

**Figure 11:**
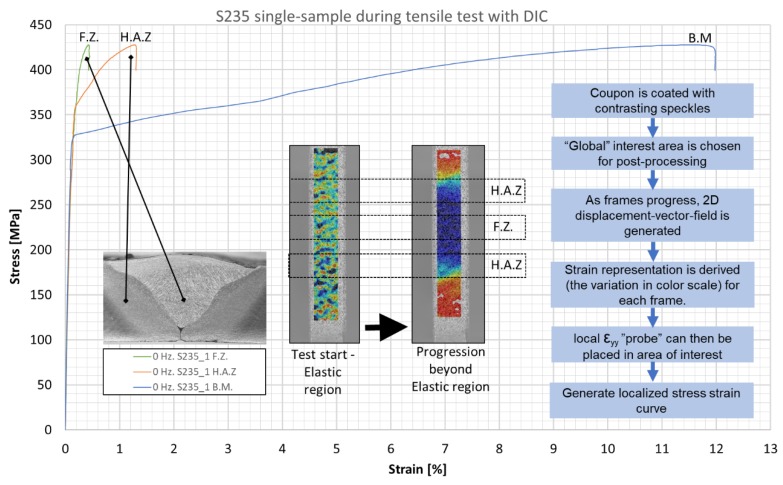
Illustration of the procedure for tensile test testing with digital image correlation (DIC). A digital “strain gage” is placed at five points along the global area of interest (FZ × 1, HAZ × 2, BM × 2). The results are averaged and three lines are plotted, one for each zone. These plots are all from the same sample, hence, the ultimate tensile strength (UTS) is that of the weakest area of the sample.

A cross-section of a weld is an intrinsically non-homogeneous material. The FZ, HAZ and BM have different mechanical properties. Unlike traditional tensile testing with strain gages or extensometers (one setup, one test), DIC allows full-field generation of local deformation fields, frame by frame, throughout the duration of the test. The result is a recorded area that can be probed digitally to produce local stress-strain behavior. In conventional tensile testing of welds, in order to achieve a specific stress-strain plot of the filler material (FZ), a large weld volume is needed (e.g., multipass welds with thermal effects) so that a dog-bone sample can be machined. HAZ characterization is even harder to implement and quite impractical. With DIC, in a single tensile test of the weld, it is possible to generate separate distinct stress-strain plots for the FZ, HAZ, and BM. Figure 11 outlines the procedure and shows typical test outputs of one sample, where a digital probe (“strain gage”) is placed on five points along the global interest area (FZ × 1, HAZ × 2, BM × 2). The colored stripes are digital image outputs of the dog-bone’s neck during the test, with momentary strain-magnitude color coding. The separate material behavior is clearly observed once the test progresses to the elastic limit of the BM (in this case). Applying a symmetry condition for HAZ and BM data, these results are averaged, and three lines are plotted for the different material zones. The plots in Figure 11 are all from the same sample and, therefore, the ultimate tensile strength (UTS) is that of the weakest area of the sample. The sample eventually failed in the BM area, thus it may be concluded that the experiment ended prematurely in the case of the HAZ and FZ.

**Figure 12:**
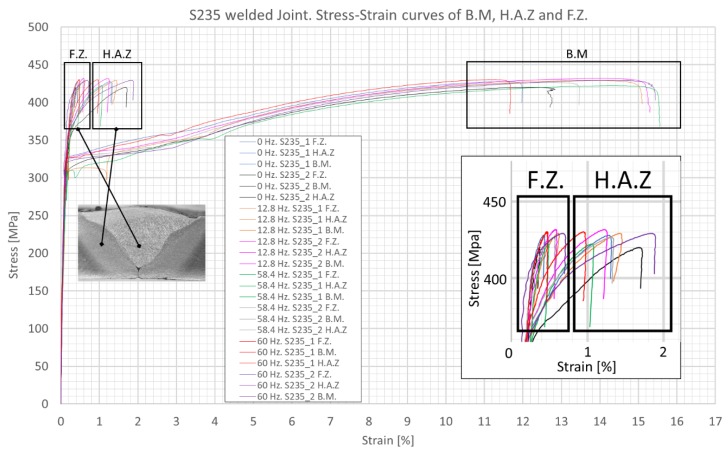
Tensile test curves for S235 steel welds, revealing the local mechanical properties of different weld zones.

**Figure 13:**
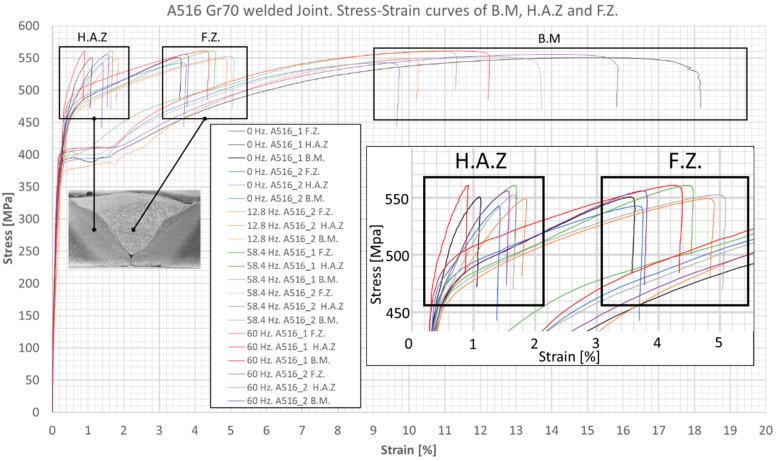
Tensile test curves for A516 steel welds, revealing the local mechanical properties of different weld zones.

Using the aforementioned procedure, the results for S235 and A516 welded samples are shown in Figure 12 and Figure 13, respectively. In all cases, necking and fracture occurred at the BM. Localized stress-strain analysis with DIC allowed for the following observations: (1) The areas of interest (e.g., FZ and HAZ) showed no quantitative difference in the mechanical stress-strain behavior between the vibrated and non-vibrated samples. (2) For the BM of S235 steel, the average yield strength was ~325 MPa and the tensile strength was higher than 410 MPa (i.e., exceeding the requirement of EN 10025 standard). (3) For the BM of A516 steel, the average yield strength was ~370 MPa and the tensile strength was higher than 530 MPa (i.e., exceeding the requirements of ASTM A516 standard). (4) The average yield strength of the weld material (ER70S-6) was 357 MPa for S235 samples and 353 MPa for A516 samples (i.e., roughly the same). (5) The tensile test of S235 steel stopped in the range of 420–440 MPa (namely, the tensile strength of the S235 BM). At these stress values, the FZ exhibited 0.4–0.6% strain, whereas the HAZ exhibited 0.9–1.9% strain. (6) The tensile test of A516 steel stopped in the range of 540–560 MPa (namely, the tensile strength of the A516 BM). At these stress values, the FZ exhibited 3.6–5.2% strain, whereas the HAZ exhibited 0.9–1.9% strain. This is in correlation with the microhardness data, suggesting a local quenching effect near the fusion line, with increased hardness. (7) The FZ (diluted filler + BM) of S235 steel had higher yield strength than that of the HAZ. (8) In contrast, the FZ of A516 steel had lower yield strength than that of the HAZ. (9) Both curves of the BM show a classic ductile behavior of mild steel. The results are as expected for these types of steels and filler wire. (10) All samples failed at the BM, far from the HAZ (as expected in a properly performed weld), with no distinct advantage for the vibrated samples.

## 4. Discussion

In the case of a simple forced vibration by deterministic harmonic excitation of a discrete linear system with a single degree of freedom (DOF), the output is a deterministic harmonic response. The relations between the structural properties and the excitation magnitude and frequency reveal interesting phenomena, as shown in Appendix A. In reality, the case of forced vibration of a continuous linear system (e.g., a structure with infinite degrees of freedom) by deterministic harmonic excitation is much more complicated and depends on structure geometry, material, location of the exciter, fixtures, type and shape of supporting dampers, etc. Different locations along the structure will vibrate with different characteristics and experience different alternating stresses in the process. Many publications on vibration-assisted welding refer to the exciting frequency as the main “input” parameter in addition to the resonant frequency. Knowing that the resonance frequency (natural 1st mode) of a certain structure is mainly a function of its geometry, the classification of the vibratory regime in relation to the weld as “resonant” or “sub-resonant” seems irrelevant. The local weld joint experiences a certain vibrational characteristic, whether it is a small weld coupon or a weld that is part of a big structure.

Residual stresses in welded structures are the cause of structural deformations and are often examined in works dealing with vibrational welding, since they are measurable (using the hole-drilling method, for example). The reduction of residual stresses is often mistakenly attributed to vibration. Whether vibration is induced during welding or post-welding (as in the case of VSR), the stress relief is not a direct result of the LVE, but rather a byproduct of the structural response that yields plastic deformations around the weld areas, thus achieving stress relief. This has been supported by References [9,10,11,12,13,14] and few cycles are enough, as demonstrated in References [11,15,16] and the experimental setups for GVE [10,14,15]. In the case of simultaneous vibration and welding, the weld area is further heated, which causes the yield strength to drop significantly, implying that under the same strain magnitude, the material will be more prone to plastic deformation. In most cases of real structures, unless a displacement-controlled mechanism is used, exciting the structure at a near-resonant frequency is the only means to produce large local strains using a small vibrator. 

Differentiation between vibration as a root cause for various phenomena and vibration as a facilitator of translations/deformations (LVE vs. GVE) must be taken into account in the experimental procedures and analytical approaches to the study of vibrational welding. Overlooking it might be the origin of some confusion regarding the “optimum” conditions (e.g., of frequency, amplitude, and direction) for producing better welds with vibratory welding. For example, in Reference [17,18,19], a large pipe-like structure was welded during vibration at 54–59 Hz. The reduction of residual stresses and structural deformation was reported. Based on the geometrical dimensions of the pipe provided in these articles, a 3D model of the pipe was constructed and a simple frequency analysis using FEA was conducted. The analysis showed that the 1st-mode resonant frequency was 58.8 Hz, which was very close to the frequency used in the experiment, implying that GVE may have been the cause for stress reduction via momentary plastic deformations.

Of the numerous articles reviewed that suggest a critical distinction between LVE and GVE, as explained before, only one work with LVE has shown substantial improvement in the mechanical properties of the weld [20,21]. The weld, in that case, was vibrated at higher frequencies (80–400 Hz) than those in the current work, and amplitudes were in the range of 5–40 µm. The results showed an increase in microhardness and tensile strength as the frequency increased.

**Figure 14:**
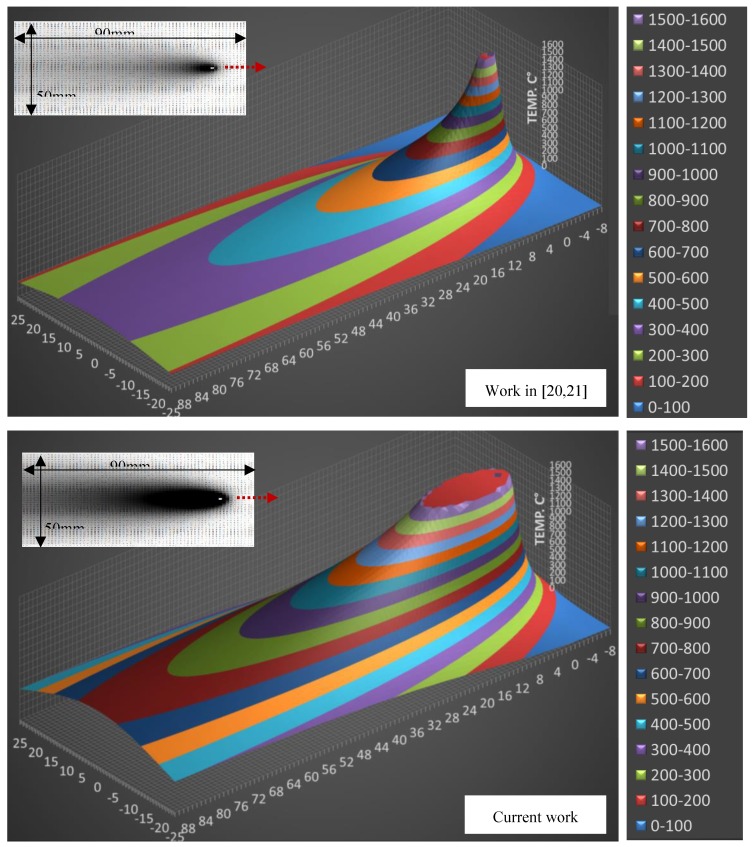
Surface temperature distributions (°C) around an advancing heat source as a function of (x, y) position. Solution of Rosenthal’s equation for 2D thin plates plotted at 1 mm intervals for 50 × 90 mm plate. Top: Based on Tewari and Shanker’s data [20,21] (weld parameters: 25 V, 135 A, 5 mm/s; the welding speed being assumed since no value was provided in those articles). Bottom: Current work welding parameters (33 V, 225 A, 4.9 mm/s). At the top left are 2D grayscales of temperature gradient: Black representing ≥ 1500 °C, white = 25 °C.

**Figure 15:**
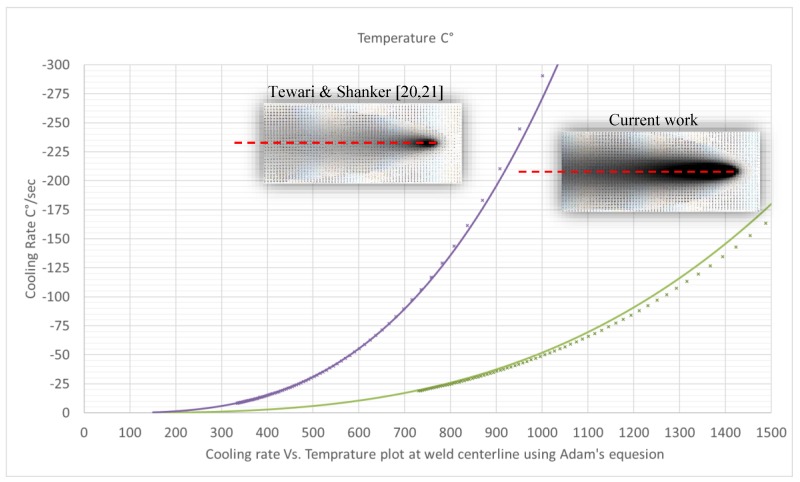
Cooling rates along the fusion zone centerline as a function of momentary temperature. Solid lines are solutions of the Adam’s equation. Symbols are numeric results from Rosenthal’s solution for a temperature distribution (Δ*T*/Δ*t*). The weld in Reference [20,21] had steeper temperature gradients and higher cooling rates than those in the current work.

In order to better understand what other factors (if any) might cause such different results, a comparative simulation of the temperature field of both works was run using Rosenthal’s equation, as shown in Figure 14. This equation along with the parameters used for solving it are given in Appendix A. Rosenthal’s equation, though conceived with simplifying assumptions, is a simple and powerful tool for qualitative visualization of the heat flow around the advancing weld. As can be seen in Figure 14, the thermal regime of the two welds was quite different. Using the same equation and plotting the cooling rates as a function of the momentary temperature along the weld centerline yields (Figure 15), it was clear that the weld in Reference [20,21] had steeper temperature gradients and higher cooling rates than those in the current work. Cooling rates within the temperature range of 800–500 °C, are about 5 to 6 times greater than those in the current work.

This comparison does not explain the different results of the two works. It does, however, emphasize the fact that there are other essential variables that need to be considered when trying to explain results simply by comparing frequency and amplitude in works involving vibrational welding. From this qualitative analysis, two points emerge: (1) A threshold frequency value may exist, at which no apparent effect occurs. The current work employed the low-frequency range of 12–60 Hz, whereas in Reference [20,21], was in the range of 80–400 Hz. (2) A threshold cooling rate magnitude (i.e., a slow cooling rate) may exist, under which no apparent effect is apparent regardless of vibration parameters. The current work involves cooling rates of 26 to 6 °C/s at temperatures of 800 °C to 500 °C, respectively. In comparison, the cooling rates in Reference [20,21] were 133 to 32 °C/s at temperatures of 800 °C to 500 °C, respectively.

As discussed before, the conditions for producing a favorable weld using vibratory welding are not yet concluded. Therefore, future empirical work should concentrate on finding the combination of frequency/amplitude/direction at which favorable reproducible results can be achieved with respect to LVE. If such a combination is found, it will then be possible to use vibrational control systems to impose such conditions on structural joints from an industrial perspective. From an analytical perspective, on the other hand, it may serve as a validation tool for a comprehensive model of solidification under vibratory conditions.

## 5. Conclusions

Mechanical and microstructural analysis of vibrated and non-vibrated steel welds allowed the determination of the following conclusions:

1. Typical microstructures of the FZ and HAZ were evident, with no distinct difference between vibrated and non-vibrated samples.
2. The microhardness values were typical of the two plates of steel and filler wire, with no distinct advantage to vibrated samples: 195 VHN for FZ (in both materials), 165 VHN for S235 CGHAZ, and 280 VHN for A516 CGHAZ.
3. The tensile test data were in accordance with typical values for the two steels and filler wire. S235 steel had an average yield strength of ~325 MPa and an average tensile strength of 410 MPa. A516 Gr. 70 steel had an average yield strength of ~370 MPa and an average tensile strength of 530 MPa. The yield strength of the weld material (ER70S-6) was 357 MPa for the S235 samples, and 353 MPa for the A516 samples. All samples failed at the metal far from the HAZ (as should be in a proper weld), with no distinct advantage to vibrated samples.
4. Tensile testing of welds using DIC proved to be an effective tool for differentiating the separate material behaviors of the different weld areas (BM, HAZ, and FZ).
5. Low-frequency rigid-body-motion vibration during welding in the normal-to-plate direction had no beneficiary effect on weld properties.
6. Nevertheless, vibrating structures while welding can, in some cases, contribute to stress relief by means of small deformations induced by the structural vibration, because the locally heated joints (with their substantially lower yield strength) experience stresses that exceed the yield strength. This procedure, however, is strongly dependent on the structure’s geometry and frequency response.

## Figures and Tables

**Table 1:** Summary of welding parameters.

Frequency (Hz)	Vertical Amplitude, Peak to Peak (mm)	Welding Speed, *v* (mm/s)	I (A)	V (V)	Net Heat Input $Q=\frac{\mathrm{VI}\eta }{\nu }\;*$ (kJ/mm)
0	0	5.22	225	33	1.138
58.4	0.25	4.71	227	33	1.273
60	0.8	4.9	230	34	1.277
12.8	0.3	4.9	226	32	1.181

^*^η = 0.8 for GMAW [7].

**Figure A1:**
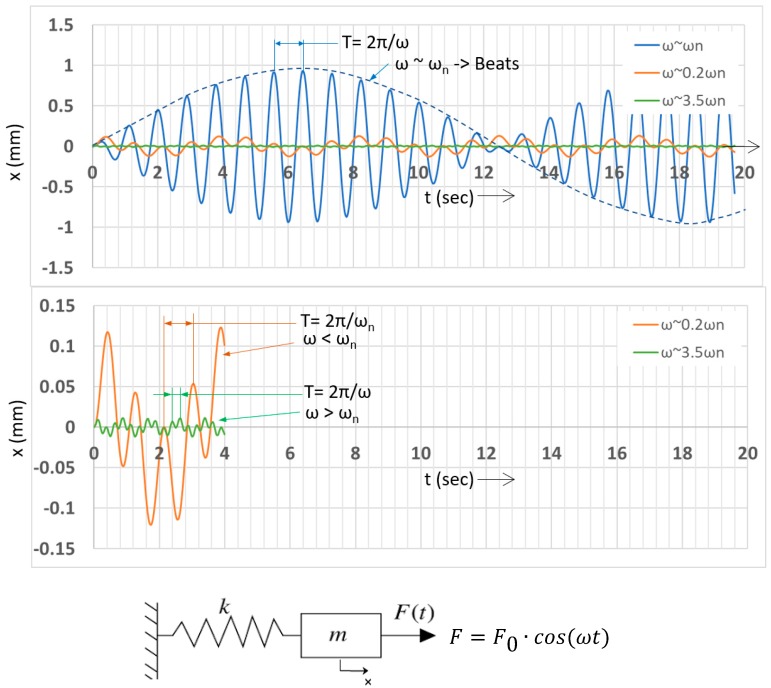
Spring and mass response to harmonic excitation (vibration). Top plot: Three different responses where the only parameter changed is the excitation frequency. The results are plotted on the same graph for *ω* ~ *ω_n_* (blue), *ω* < *ω_n_* (orange), and *ω* > *ω_n_* (green). Bottom plot: Showing only the *ω* < *ω_n_* (orange) and the *ω* > *ω_n_* (green) plots with corrected ordinate (abscissa is the same).

## Appendix A

### Appendix A.1. Various Responses to Excitation in a Single DOF System

The following example is given in order to illustrate how a harmonic excitation of a structure can produce various forms of structural responses depending on structural properties in relation to the excitation frequency. For simplicity, the example illustrated in Figure A1 is of a simple spring and mass system, for which the equation of motion is:

(A1) $$m\ddot{x}+k\ddot{x}=F_{0}\cos\left(\omega t\right)$$

The solution is given in detail in Reference [22], pp. 263–269, and is in the form of:

(A2) $$x_{\left(t\right)}=\left(x_{0}-\frac{F_{0}}{k-{m\omega }^{2}}\right)\cos\left(\omega_{n}t\right)+\left(\frac{{\dot{x}}_{0}}{\omega_{n}}\right)\sin\left(\omega_{n}t\right)+\left(\frac{F_{0}}{k-{m\omega }^{2}}\right)\cos\left(\omega t\right)$$

For initial conditions of *x*_0_ = 0 and ${\dot{x}}_{0}=0$:

(A3) $$x_{\left(t\right)}=\left(\frac{F_{0}}{k-{m\omega }^{2}}\right)\left[\cos\left(\omega t\right)-\cos\left(\omega_{n}t\right)\right]$$

where *x**_(t)_* is mass location (mm), *ω**_n_* is the natural frequency (=$\sqrt{k/m}$, rad/s), *ω* is the excitation frequency (rad/s), *k* is the spring constant (N/mm), *m* is the mass (kg), and *F*_0_ is the excitation amplitude (N).

The overlapping plots of Figure A1 are the solutions to Equation (A3); the only variation is the excitation frequency, *ω*. The same scale on the abscissa (time) is used in order to get a sense of the magnitude of change in amplitudes with respect to change in excitation input frequency. In vibrational welding applications of real structures, the deterministic harmonic excitation is that of a continuous system with infinite degrees of freedom, and yields a more complicated harmonic output.

### Appendix A.2. Temperature Distribution Around a Moving Weld

Equation (A1) is Rosenthal’s equation for the temperature distribution for 2D thin plates (*z* = 0). Simplifying assumptions of this equation are: (1) Steady-state heat flow; (2) point heat source; (3) negligible heat of fusion; (4) constant thermal properties; (5) no heat losses from the workpiece surface; (6) no convection in the weld pool. One gets:

(A4) $$\frac{\left(T-T_{0}\right)2\pi d\kappa }{\eta Q}=e^{\frac{\nu x}{2\alpha }}K_{0\left(\frac{\nu r}{2\alpha }\right)}$$

where *T* is current temperature (*K*), *T*_0_ is initial temperature/preheat (*K*), *d* is the plate thickness (mm), *k* is the thermal conductivity (J/(mm⋅s⋅K), *Q* is the welding heat input (W), *r* is radius vector coordinates ($\sqrt{x^{2}+y^{2}}$), where *x* is the weld direction and *y* is the transverse direction, both in (mm)), *ν* is the welding speed (mm/s), *α* is the thermal diffusivity (mm^2^/s), *η* is the welding heat source efficiency, and *K*_0_ is a modified Bessel function of 2nd type, zero-order, with argument $\left(\frac{\nu r}{2\alpha }\right)$.

The temperature distribution is thus given by:

(A5) $$T=\frac{{\eta QK}_{0\left(\frac{\nu r}{2\alpha }\right)}e^{\frac{\nu x}{2\alpha }}}{2\pi d\kappa }+T_{0}$$

The interested reader should refer to Reference [7] (pp. 48–52) and [23] (pp. 29–54) for further explanations, as well as for Adam’s Equation for cooling rates along the weld’s centerline.
